# Supplementary material for: Patterns of Intron Gain and Loss in Fungi
Source: PLoS Biol. 2004 Nov 30;2(12):e422. doi: 10.1371/journal.pbio.0020422 (PMC532390; doi:10.1371/journal.pbio.0020422)
Supplement: Table S1 — Also available at http://genes.mit.edu/NielsenEtAl/. (4.3 MB ZIP). [file pbio.0020422.st001.zip › NielsenEtAl/html/494.html]

AN5701.1.NCU05548.1.MG09766.1.FG08338.1


```
 CLUSTAL W (1.82) Multiple Sequence Alignments - Introns Inserted


Sequence 1: NCU05548.1	379 aa
Sequence 2: FG08338.1	365 aa
Sequence 3: MG09766.1	383 aa
Sequence 4: AN5701.1	362 aa
Alignment Length: 384 aa
Number Identitical Residues: 229 aa
Alignment Score (without introns) 10032


MG09766.1 	-MSNGTNMVPAQVVFDDTRV1LGQDPLIPPQILMEEIPI~TKAALETVVRGRQEAVACVI
NCU05548.1	--MVGPDMV--ALRADDTRV1EGQDPLIPPALLISEIPM~TDAALETVVKGRKEAVGVIM
FG08338.1 	MPSATIDMS--TIAADDTRV1LGQDPLIPPALLTSEIPL~PEKATNTVVKGRQDAADIVL
AN5701.1  	-------MS---GQNEDTRV1LGYDPLLSPEFVQSEIPS0NEHSIPTVRSGRNQAVEIIE
          	       *       :****  * ***:.* :: .***   . :  **  **::*.  : 

MG09766.1 	GRDDRLLVIVGPCSIHDPAAALEYCQRLKALADKLSDDLCIVMRAYLEKPRTTVGWKGLI
NCU05548.1	GKDDRLLVVVGPCSLHDPATALEYCSRLKALSEKLKDDLVIVMRAYLEKPRTTVGWKGLI
FG08338.1 	GQSDRLLVVVGPCSIHDPATAQEYAARLKEVSDRLDKDLCIVMRAYLEKPRTTVGWKGLI
AN5701.1  	QRDDRLLVVVGPCSIHDPETALEYATRLKELAARLSSDLCVIMRAYLEKPRTTVGWKGLI
          	 :.*****:*****:*** :* **. *** :: :*..** ::******************

MG09766.1 	NDPDIDQTFKINKGLRISRQIFCDLTSAGMPIASEMLDTISPQFLADLISVGAIGARTTE
NCU05548.1	NDPDIDSSFKINKGLRISRQLFCDLTSAGMPIASEMLDTISPQFLADFISVGAIGARTTE
FG08338.1 	NDPDIDNSFKINKGLRVSRQLFVDLTTQGLPIATEMLDTISPQFLADCISVGAIGARTTE
AN5701.1  	NDPDIDESYQINKGLRVSRKLYADLTGMGMPIASEMLDTISPQYMADLISLGAIGARTTE
          	******.:::******:**::: ***  *:***:*********::** **:*********

MG09766.1 	SQLHRELASGLSFPVGFKNGTDGNLGVAIDAIGAAASKHSFMGVTKQGLAAITRTAGNQH
NCU05548.1	SQLHRELASGLSFPVGFKNGTDGNLGVAIDAIGAAAAKHHFMGVTKQGLAAITRTKGNEH
FG08338.1 	SQLHRELASGLSFPVGFKNGTDGSLNVAIDAIGAAAAQHHFMGVTKQGLAAITRTKGNEH
AN5701.1  	SQLHRELASGLSFPIGYKNGTDGNLTVAIDAIGAAAHPHRFLGVTKQGLAAITKTAGNPH
          	**************:*:******.* **********  * *:***********:* ** *

MG09766.1 	GFVILRGGTKGPNFDKASVQAAKETLVQKGQRQAIMIDCSHG1NSNKDHRNQPKVAKVIG
NCU05548.1	GFVILRGGTKGPNYDKENVQAAKETLIKKGQKLAIMVDCSHG1NSNKDHRNQPKVAKVVA
FG08338.1 	CFVILRGGTKGTNFDKDSVQNAKKVLQDKKQKEAIMIDCSHG1NSSKDHRNQPKVAKVVG
AN5701.1  	GFVIMRGGNKGTNYDRESIQGAREALRGKKQREVLMVDCSHG~NSKKNHRNQPLVAKEVA
          	 ***:***.**.*:*: .:* *::.*  * *: .:*:***** **.*:***** *** :.

MG09766.1 	DQLREGERSIIGVMIESNINEGNQKVPAEGPSGLKKGVSITDACIDWDSTVAVLEDLADA
NCU05548.1	DQIREGEKAIIGVMIESNINEGNQKVPAEGPSALKKGVSITDACIDWESTVSTLEELAEA
FG08338.1 	EQLREGEKAIIGVMIESNIGEGNQKVPAEGPAALKRGVSITDACIGWEDTVTVLEDLADA
AN5701.1  	DQLREGQDAIIGVMIESNINEGNQKVPPEGPSGLKKGVSITDACIDWETTVTVLEDLADA
          	:*:***: :**********.*******.***:.**:*********.*: **:.**:**:*

MG09766.1 	VRARRSLYPNDRNVDREERLATPLEED
NCU05548.1	VRERREVN-GGLASGVATPKPNPLEED
FG08338.1 	VRTRRKLN-TA----------------
AN5701.1  	VRARRAVKSKANGTA------------
          	** ** : .
```
